# Video-based Learning Versus Traditional Method for Preclinical Course of Complete Denture Fabrication

**Published:** 2015-03

**Authors:** Amir Fayaz, Azita Mazahery, Mohammad Hosseinzadeh, Samane Yazdanpanah

**Affiliations:** 1 Dept. of Prosthodontics, School of Dentistry, Shahid Beheshti University of Medical Science, Tehran, Iran;; 2 Dept. of Prosthodontics, Dental Branch, Islamic Azad University, Tehran, Iran;; 3 Dept. of Medical Education, Shahid Beheshti University of Medical Science, Tehran, Iran;; 4 Dept. of Oral and Maxillofacial Radiology, Dental Branch, Rafsanjan University, Kerman, Iran;

**Keywords:** Dental Education, Video-based Learning, Post-test, Pre-test, Complete Denture

## Abstract

**Statement of the Problem:**

Advances in computer science and technology allow the instructors to use instructional multimedia programs to enhance the process of learning for dental students.

**Purpose:**

The purpose of this study was to determine the effect of a new educational modality by using videotapes on the performance of dental students in preclinical course of complete denture fabrication.

**Materials and Method:**

This quasi-experimental study was performed on 54 junior dental students in Shahid Beheshti University of Medical Sciences (SBMU). Twenty-five and 29 students were evaluated in two consecutive semesters as controls and cases, respectively for the same course. The two groups were matched in terms of "knowledge about complete denture fabrication" and "basic dental skills" using a written test and a practical exam, respectively. After the intervention, performance and clinical skills of students were assessed in 8 steps. Eventually, a post-test was carried out to find changes in knowledge and skills of students in this regard.

**Results:**

In the two groups with the same baseline level of knowledge and skills, independent T-test showed that students in the test group had a significantly superior performance in primary impression taking (*p*= 0.001) and primary cast fabrication (*p*= 0.001). In terms of anterior teeth set up, students in the control group had a significantly better performance (*p*= 0.001).

**Conclusion:**

Instructional videotapes can aid in teaching fabrication of complete denture and are as effective as the traditional teaching system.

## Introduction

A successful medical education system should enable the instructors to address the learners’ needs and understand the variations in teaching styles and approaches.[1] The potential role of multimedia in learning has long been a matter of debate among researches. Recent changes in dental and medical school curricula have also added to the value of computer-assisted learning (CAL).[2] In a review of CAL, it was concluded that this method provides clinicians with better decision-making skills.[3] Numerous studies have evaluated the impact and role of e-learning in medical and dental education systems. Many researchers have found no significant difference between e-learning and conventional class-room teaching,[2-6] which means that the performance of students was the same in traditional (lecture-based) and CAL. However, it has been suggested that CAL may successfully replace traditional learning strategies.[7] Some studies consider e-courses or e-learning as a supplement or complement to traditional teaching methods.[8-9] Several studies discussed the advantages of this new educational system and reported that this system can be an additional resource for students,[10] may help in instruction of fixed prosthodontics,[11] assist students in developing skills more efficiently,[12-13] as well as having a significant impact on skills scores of students.[14] Other studies revealed that a higher percentage of students preferred e-learning and had a positive perception towards it.[15-18] In another study, the computer-assisted and computer-assisted plus text-based learning groups gained significantly more knowledge than the text-based learning group.[19]

However, controversy still exists in this respect. For instance, one study reported significantly higher learning retention in trainees through lecturing rather than e-learning.[20] A study by Walmsley *et al.* indicated differences in attitudes of the staff and students towards the use of internet as a resource for dental education.[21] Another study showed that dentists, similar to dental students, disliked reading electronic textbooks.[22] Responses to the survey revealed some disagreements between administrators and information technology specialists.[23] Application of new technology to promote learning in academic contexts is rapidly growing and relevant experiences of researchers and the results of research projects should be incorporated in the educational curriculum. E-curriculum planners should pay close attention to implementation problems,[24] and distance learning should be organized with a mixture of audio and video input.[25] However, it has been suggested that CAL should not replace traditional education.[26] Clearly, this topic needs further scrutiny in complex theoretical and practical (clinical) contents. Complete denture fabrication is a lengthy preclinical course in undergraduate dental curriculum covering all phases of denture fabrication from primary impression to finishing and polishing. Under these circumstances, it seems necessary to analyze and compare the students’ skills between the two groups in every step of the procedure. Studies comparing the results of different instruction techniques in this respect are scarce and to the best of our knowledge, no study has compared scores gained by students from each phase of fabrication in the two groups of conventional and multimedia instruction.

The purpose of this study was to assess and compare the effectiveness of standard lecture with computer assisted learning using an educational CD ROM developed for preclinical course of denture fabrication at the School of Dentistry, SBMU. The aim was to clearly demonstrate differences or similarities between the two groups in each stage of practical course from primary impression to complete arrangement of teeth. 

## Materials and Method

This quasi-experimental study was conducted on 54 students in two consecutive semesters at School of Dentistry, SBMU. Twenty-five students in the first semester and 29 students in the second semester were evaluated as controls and cases, respectively. No age or sex limit was set. The understudy subjects all had taken the preclinical complete denture fabrication course. 

The traditional educational content included familiarization of students with the materials and instruments used in the complete denture preclinical course for fabrication of a complete denture set including primary impression taking, fabrication of primary cast, fabrication of special tray, border molding, final impression taking, master cast, record base and wax rim fabrication, mounting the master casts on the articulator, and anterior and posterior teeth set up. The traditional course was held during 30 sessions. In the conventional classroom learning, two instructors from removable prosthodontics department trained the students. The trainees could consult their instructors during the two sessions that were held twice a week if they had any problem. 

In the new experimental method, all the above-mentioned steps were taught to the students with the use of videotapes and other supplemental educational tools. The process of learning in this method was supervised by an instructor and a technician working in the removable prosthodontics department. All students in the test group had access to a personal computer in the class and watched the related videotapes throughout the sessions. Instructors also participated in these sessions. Similar to conventional learning, 30 sessions were held. It should be mentioned that students had to practice only during the sessions and were not allowed to take their work out of the class. 

*Assessment of the baseline knowledge of subjects*

 Since students from two different classes were selected as the case and control groups, at the beginning we had to make sure that the two groups had the same level of baseline knowledge about complete denture fabrication. Therefore, at baseline, a written exam (exam I) was taken from students in both groups containing 10 questions. The reliability of the exam was approved by a faculty member of School of Dentistry, SBMU. The students in both groups were given ten minutes to answer the questions. By doing so, the two groups were matched in terms of their baseline information regarding this topic. 

*Assessment of practical skills*

 In order to evaluate and compare the practical skills of students in the two groups, a practical dental anatomy exam was held (exam II) that included carving a maxillary canine tooth out of a wax block. Assessment of the scores of exam II was done by one of the operative dentistry faculty members.  

*Assessment of level of knowledge about the complete denture fabrication*

In the next phase, in order to assess the knowledge of students in both groups about complete denture fabrication before the initiation of the preclinical course, a written exam was taken containing 9 questions. The reliability of the test had been confirmed by a group of faculty members (exam III, pretest). Students had 10 minutes to answer the questions.

*Educational methods*

For the first group of students (control group), teaching was performed traditionally by instructors in all steps and demonstrations of each step were done in form of live teaching. For the second group of students (case group), teaching was done based on multimedia educational system (that had been prepared in advance) by using a CD. The CD was prepared by the prosthodontics faculty members (removable section) and included teaching of A-Z steps of complete denture fabrication on a model.

*Preparation of an educational CD*

For education of students in the intervention group, an instructional CD regarding preclinical complete denture fabrication was prepared by a group of faculty members in the Department of Removable Prosthodontics and IT Department of SBMU. After preparation of each part of the educational film, its contents were evaluated by the instructors. The instructional video depicted the step-by-step procedures of preclinical fabrication of a complete denture (familiarization of students with the materials and instruments, primary impression taking, primary cast fabrication, special tray fabrication, master cast fabrication, record base and wax rim fabrication, mounting the master casts on the articulator, and anterior and posterior teeth set up). The reliability of this film was confirmed by the faculty members of the Department of Removable Prosthodontics and IT Department of SBMU.

Students in the experimental group had access to this video only during their preclinical class time and were not allowed to take it out. This CD replaced the traditional learning in the test group. 

*Assessment of the practical skills of students for fabrication of complete denture*

After completion of both educational methods, in order to assess the practical skills of students for complete denture fabrication, performance of students in primary impression taking, fabrication of primary cast, fabrication of special tray, fabrication of master cast, mounting the master casts on the articulator, fabrication of record base and wax rim, and anterior and posterior teeth set up was scored in a score sheet especially designed for this purpose by the department of removable prosthodontics. This score sheet was considered as exam IV. It should be mentioned that reviewers who assessed the above-mentioned phases were blinded to the study. 

*Assessment of the final knowledge of students about complete denture fabrication*

After completion of the course in the two groups, a written exam was held with 23 questions (exam V) in order to assess and compare the changes in knowledge of students in the two groups. The reliability of the test was confirmed by faculty members of the department of removable prosthodontics. Two tests were held separately for each group and the students were supposed to answer the questions in 35 minutes. 

The quality of third and fifth exams had to be assessed in terms of level of difficulty. Thus, 10 senior students that had a GPA score over 3.5 were selected to take the exams. They were given the same time periods (as the test and control groups) to answer the questions and the results were evaluated. Based on their test results and according to the recommendations of the faculty members, questions of the third and fifth exams were corrected.

*Data analysis*

Descriptive and analytical statistics were used to test the hypotheses and compare the results. In order to test the normal distribution of data, one-sample Kolmogorov-Smirnov test was used. For the assessment of inter-rater reliability in 8 domains of the practical skills test (exam IV), intraclass correlation coefficient (ICC) was calculated. After controlling the small baseline differences, analysis of covariance (ANCOVA) was applied to compare the two groups in terms of their obtained scores in different domains (exams IV and V). For pairwise comparison of the two groups in terms of baseline variables, independent T-test was used. SPSS software version 15 was used for statistical analysis and *p*< 0.05 was considered statistically significant.

This study was approved by the Ethics Committee of SBMU. Written informed consent was obtained from all students before their enrollment in the study. Scores obtained by students in the mentioned tests had no effect on their final score of the course. 

## Results

Data obtained for quantitative variables in all practical steps (impression taking, primary cast fabrication, etc.) had normal distribution with the minimum odds ratio of 0.145. Thus, parametric tests were applied. T-test was used to compare the two groups in exams I, II and III. No significant differences were found between the two groups in exams I (*p*= 0.059), II (*p*=0.263) or III (*p*= 0.625). Since exam II had two parts, to reach a final score, ICC was calculated to be 0.736, which revealed that the scores were close. Thus, the mean of values was calculated to reach a final score (Table 1). It indicated that the two groups were successfully matched in terms of their theoretical and practical skills at baseline. Although the two groups had no statistically significant difference in important baseline variables because the means and standard deviation (SD) in the two groups were slightly different, ANCOVA was applied to eliminate the small differences and for more precise comparison of the two groups.

**Table 1:** Statistical indices for the three test scores obtained by the two groups of students

**Variables**	**Traditional instruction**		**Multimedia instruction**		**P-value**
	**Mean**	**SD**	**Mean**	**SD**	
Test I	53.20	24.62	42.07	17.60	0.059
Test II	284.20	45.25	268.27	56.44	0.263
Test III	150.40	48.69	157.24	52.84	0.625

The results of the fourth exam (test IV, practical exam) were as follows:   

Primary impression taking: Three assessments were done for this variable. To reach a final value, ICC was first calculated to be 0.698, which was acceptable. Thus, the mean of three scores was used for comparison of the two groups revealing a significant difference (*p*= 0.001). Students in the test group had a significantly superior performance in this regard compared with controls.

Primary cast fabrication: In order to reach the final value, ICC was calculated to be 0.818, which was acceptable and the mean of three scores was used for comparing the two groups. Students in the test group performed significantly better than controls in this regard (*p*= 0.001).

Special tray fabrication: ICC was calculated to be 0.721, which was within the acceptable range and the comparison of the two groups revealed no statistically significant difference in this respect (*p*= 0.639).

Master cast fabrication: ICC was calculated to be 0.731 and the mean of three scores was used to compare the two groups. Students in the test group had a slightly better performance, which was not statistically significant (*p*= 0.225).

Record base and wax rim: ICC was calculated to be 0.774. The mean of three scores was calculated. Students in the test group had a slightly better performance, which was not statistically significant (*p*= 0.986).

Mounting master casts on the articulator: ICC was calculated to be 0.526. The mean of three scores was calculated and the difference between the two groups was not statistically significantly different in this respect (*p*= 0.706). Anterior teeth set up: ICC was calculated to be 0.581.

The mean of three scores was calculated. After adjusting for baseline variables, the test group showed significantly lower performance than controls (*p*= 0.001).

Posterior teeth set up: ICC was calculated to be 0.693, which was within the acceptable range. The two groups showed no statistically significant difference in this regard (*p*= 0.773).

**Figure 1:**
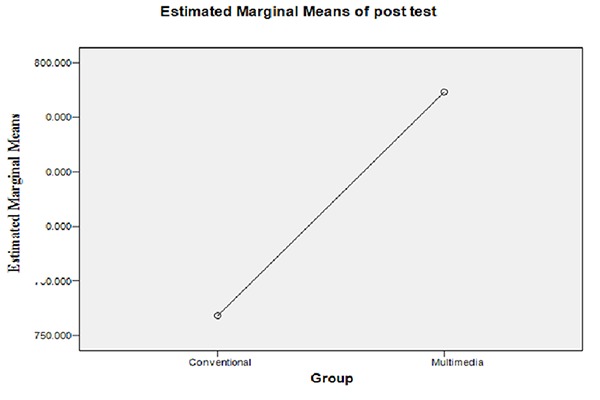
Comparing the scores of test V between the two groups

The obtained score in the fifth exam was 753.62±33.92 in the control and 794.61± 31.38 in the test group and the difference between the two was not statistically significant (Table 2 and Figure 1).

**Table 2:** Statistical indices for scores obtained by students in the two groups in tests IV and V

**Variables**	**Traditional instruction**		**Multimedia instruction**		**P-value**
	**Mean**	**SD**	**Mean**	**SD**	
Primary impression taking	0.097	0.029	0.129	0.023	0.001
Primary cast fabrication	0.741	0.221	0.924	0.158	0.001
Special tray fabrication	1.431	0.206	1.437	0.282	0.639
Master cast fabrication	0.966	0.143	1.011	0.127	0.225
Record base and wax rim fabrication	2.231	0.338	2.259	0.373	0.986
Mounting the master casts on the articulator	0.302	0.043	0.299	0.033	0.706
Anterior teeth set up	1.894	0.231	1.628	0.267	0.001
Posterior teeth set up	2.966	0.486	2.919	0.481	0.773
Test V	753.617	33.916	794.606	31.376	0.391

## Discussion

Advancements in science and technology now allow for the application of multimedia in educational curricula.[12, 27] In this study, a multimedia courseware package in preclinical complete denture fabrication was developed. It is worth noting that preclinical education recording was performed under the supervision of prosthodontic group (removable section), by using the audio and video center of the dental school. A senior professor along with an experienced dental technician who was supervising dental students and technicians helped us in clinical and laboratory procedures. Recording was performed via three simultaneous cameras and edition of the finished movies was done in presence of the authors. Students had to watch the related portion of movie during each class. Then, 29 subjects in the test and 25 in the control groups were evaluated. To compare the number of recruited samples in our study with other studies, Aly *et al.*[2] appraised 26 subjects, besides, Plasschaert *et al.*,[7] and Stern *et al.*[14] evaluated 28, and 168 subjects in their studies, respectively.

Since in this study the test and control groups were from different educational years, for matching the groups, three pre-tests were held to determine the students’ baseline knowledge, and practical skills in general and about complete denture fabrication in particular. This phase had been skipped in many similar studies. 

Naturally, students have different learning abilities and the instruction system should be available for all students with different abilities. Qayumi *et al.*[19] in their study on two medical universities evaluated the efficacy of three different educational modalities: computer-assisted, text-based, and a combination of both learning systems. Comparison of 2 control and 3 test groups revealed significant improvement in learning of students. In terms of knowledge measure, progress of students in the computer-assisted and computer and text learning was significantly higher than that of the students in text-based group. Learning of students with better educational background was independent of the study method, but students with poorer scores showed a better learning after using computer. In the mentioned study, the students’ baseline knowledge in the 4 groups was not assessed. In a study by Ludlow and Platin,[16] a modified crossover design was used to reduce the confounding effect of different baseline knowledge levels of students. Aly *et al.*[2] changed the groups at the end of semester and tried to reduce this effect. In another study, Abutarbush *et al.*[18] evaluated the efficacy of a self-learning computer module and reported that learning was significantly enhanced in the test group by using this method. However, no baseline assessment was done on the knowledge of students. Teasdale and Sheikh[13] concluded that knowledge of medical and dental students who used an instructional computer module on geriatric oral health was significantly higher than that of controls. In their study, the students’ baseline knowledge was not assessed either. In the current study, the baseline knowledge of the two groups was compared with pretests I (*p*= 0.059), II (*p*= 0.263), and III (*p*= 0.625), which revealed no significant difference in this respect.

Exam IV revealed the test group had a significantly better performance in terms of taking primary impression (ICC=0.698, *p*= 0.001) and the primary cast fabrication (ICC=0.818, *p*= 0.001). Thus, it seems that learning through videotapes was more efficient than traditional learning. The relative easiness of these phases may be responsible for greater efficacy of videotape learning. These results were in accordance with those found by Cecilia *et al.*[11] who evaluated preclinical denture performance of the students who took fixed prosthodontics course. However, the two groups were not matched at baseline in their study.

In the current study, the two groups had no significant difference in special tray fabrication (ICC=0.721, *p*= 0.693), master cast fabrication (ICC=0.731, *p*= 0.225), record base and wax rim fabrication (ICC=0.774, *p*= 0.986), mounting the master casts on the articulator (ICC=0.526, *p*= 0.706), and posterior teeth set up (ICC=0.693, *p*= 0.773). The opinions of judges were not always exactly the same but the obtained ICCs were within the acceptable range. Although the baseline knowledge of students assessed by pretests I and II was almost the same, better results were obtained in the test group. In total, it seems that use of educational videotapes in preclinical complete denture course was as effective as the traditional educational system. This study showed that instructional multimedia can replace the constant presence of instructors and since these videos have the ability to depict all the details, they can significantly improve the students’ knowledge. This finding is in agreement with the results of Aly *et al.*[2] In their study, Plasschaert* et al.*[7] evaluated the efficacy of a multimedia interactive tutorial program for learning endodontic problem –solving without any baseline assessment and found no difference between the test and control groups.

In our study, students in the traditional learning group performed significantly better than the test group in terms of anterior teeth set up (ICC=0.581, *p*< 0.001), which indicates that constant presence of an instructor is more efficacious than educational multimedia in this respect. However, it should be noted that students had access to the video only during the classroom sessions and anterior teeth set up is among the most difficult steps in complete denture fabrication and requires great amount of precision. According to McCann *et al.*,[28] about two-thirds of the students under e-teaching found college e-resources effective for learning but they preferred printed text and liked e-teaching as a supplement, not as a replacement for traditional lectures. These findings convinced the authors of the current study to use e-learning as a complimentary method to enhance dental education.

Obviously, having baseline knowledge about a topic can better prepare students for an educational video on that specific subject. Brownel *et al.*[20] compared lecture and e-learning in new graduates and experienced dental professionals and found that e-learning was more effective in experienced dental professionals while the situation was reverse for new graduates. In terms of maintaining the gained knowledge, Stern *et al.*[14] revealed that the students’ knowledge level decreased one year after the intervention but the skills did not. Equipping the educational environment with high-tech teaching devices can further encourage and engage students in educational activities, for instance, holding conferences and seminars can improve their clinical performance. This study showed that many students generally liked e-learning and it seems that it could be more effective when combined with face-to-face teaching. Comprehensive clinical education increases the number of skilled dentists offering better health care services to the public. However, similar studies with a larger sample size are required to generalize the results. Furthermore, in this study, the students had access to the video only during the classroom sessions. Thus, in future studies, it is recommended to provide students the chance of constant access to the educational videos through the e-learning courses.

## Conclusion

Within the limitations of this study, instructional films could be successfully used to reduce the need for constant presence of mentors or as a complement to the traditional educational systems in preclinical course of complete denture fabrication. The instructional multimedia was as effective as the traditional learning system, although traditional learning group performed significantly better than the test group in terms of anterior teeth set up. 

## References

[1] Vaughn L, Baker R (2001). Teaching in the medical setting: balancing teaching styles, learning styles and teaching methods. Med Teach.

[2] Aly M, Elen J, Willems G (2004). Instructional multimedia program versus standard lecture: a comparison of two methods for teaching the undergraduate orthodontic curriculum. Eur J Dent Educ.

[3] Schittek M, Mattheos N, Lyon HC, Attström R (2001). Computer assisted learning. A review. Eur J Dent Educ.

[4] Nance ET, Lanning SK, Gunsolley JC (2009). Dental anatomy carving computer-assisted instruction program: an assessment of student performance and perceptions. J Dent Educ.

[5] Howerton WB, Enrique PR, Ludlow JB, Tyndall DA (2004). Interactive computer-assisted instruction vs. lecture format in dental education. J Dent Hyg.

[6] Bissell V, McKerlie RA, Kinane DF, McHugh S (2003). Teaching periodontal pocket charting to dental students: a comparison of computer assisted learning and traditional tutorials. Br Dent J.

[7] Plasschaert AJ, Cailleteau JG, Verdonschot EH (1997). The effect of a multimedia interactive tutorial on learning endodontic problem-solving. Eur J Dent Educ.

[8] Gupta B, White DA, Walmsley AD (2004). The attitudes of undergraduate students and staff to the use of electronic learning. Br Dent J.

[9] Healy DG, Fleming FJ, Gilhooley D, Felle P, Wood AE, Gorey T (2005). Electronic learning can facilitate student performance in undergraduate surgical education: a prospective observational study. BMC Med Educ.

[10] Eynon R, Perryer G, Walmsley AD (2003). Dental undergraduate expectations and opinions of Web-based courseware to supplement traditional teaching methods. Eur J Dent Educ.

[11] Aragon CE, Zibrowski EM (2008). Does exposure to a procedural video enhance preclinical dental student performance in fixed prosthodontics?. J Dent Educ.

[12] Silverdale N, Katz J (2005). The impact of a distance learning death and dying course: an analysis of student self-reported changes. Nurse Educ Today.

[13] Teasdale TA, Shaikh M (2006). Efficacy of a geriatric oral health CD as a learning tool. J Dent Educ.

[14] Stern DT, Mangrulkar RS, Gruppen LD, Lang AL, Grum CM, Judge RD (2001). Using a multimedia tool to improve cardiac auscultation knowledge and skills. J Gen Intern Med.

[15] Gallagher JE, Dobrosielski Vergona KA, Wingard RG, Williams TM (2005). Web-based vs. traditional classroom instruction in gerontology: a pilot study. J Dent Hyg.

[16] Ludlow JB, Platin E (2000). A comparison of Web page and slide/tape for instruction in periapical and panoramic radiographic anatomy. J Dent Educ.

[17] Howerton WB Jr, Platin E, Ludlow J, Tyndall DA (2002). The influence of computer-assisted instruction on acquiring early skills in intraoral radiography. J Dent Educ.

[18] Abutarbush SM, Naylor JM, Parchoma G, D Eon M, Petrie L, Carruthers T (2006). Evaluation of traditional instruction versus a self-learning computer module in teaching veterinary students how to pass a nasogastric tube in the horse. J Vet Med Educ.

[19] Qayumi AK, Kurihara Y, Imai M, Pachev G, Seo H, Hoshino Y (2004). Comparison of computer-assisted instruction (CAI) versus traditional textbook methods for training in abdominal examination (Japanese experience). Med Educ.

[20] Browne L, Mehra S, Rattan R, Thomas G (2004). Comparing lecture and e-learning as pedagogies for new and experienced professionals in dentistry. Br Dent J.

[21] Walmsley AD, White DA, Eynon R, Somerfield L (2003). The use of the Internet within a dental school. Eur J Dent Educ.

[22] Bates ML, Strother EA, Brunet DP, Gallo JR 3rd (2012). Electronic textbooks as a professional resource after dental school. J Dent Educ.

[23] Hillenburg KL, Cederberg RA, Gray SA, Hurst CL, Johnson GK, Potter BJ (2006). E-learning and the future of dental education: opinions of administrators and information technology specialists. Eur J Dent Educ.

[24] Hendricson WD, Panagakos F, Eisenberg E, McDonald J, Guest G, Jones P (2004). Electronic curriculum implementation at North American dental schools. J Dent Educ.

[25] Mattheos N, Nattestad A, Schittek M, Attström R (2001). A virtual classroom for undergraduate periodontology: a pilot study. Eur J Dent Educ.

[26] Oakley M, Spallek H (2012). Social media in dental education: a call for research and action. J Dent Educ.

[27] Donnelly AB, Agius RM (2005). The Distance Learning Courses in Occupational Medicine--20 years and onwards. Occup Med (Lond).

[28] McCann AL, Schneiderman ED, Hinton RJ (2010). E-teaching and learning preferences of dental and dental hygiene students. J Dent Educ.

